# Asthmatics with exacerbation during acute respiratory illness exhibit unique transcriptional signatures within the nasal mucosa

**DOI:** 10.1186/gm520

**Published:** 2014-01-17

**Authors:** Peter McErlean, Sergejs Berdnikovs, Silvio Favoreto, Junqing Shen, Assel Biyasheva, Rebecca Barbeau, Chris Eisley, Andrea Barczak, Theresa Ward, Robert P Schleimer, David J Erle, Homer A Boushey, Pedro C Avila

**Affiliations:** 1 Division of Allergy-Immunology, Department of Medicine, Northwestern University Feinberg School of Medicine, Chicago, IL 60611, USA; 2 Sandler Asthma Basic Research (SABRE) Center Functional Genomics Core Facility, University of California San Francisco, San Francisco, CA, USA; 3 Cardiovascular Research Institute, University of California San Francisco, San Francisco, CA, USA

## Abstract

**Background:**

Acute respiratory illness is the leading cause of asthma exacerbations yet the mechanisms underlying this association remain unclear. To address the deficiencies in our understanding of the molecular events characterizing acute respiratory illness-induced asthma exacerbations, we undertook a transcriptional profiling study of the nasal mucosa over the course of acute respiratory illness amongst individuals with a history of asthma, allergic rhinitis and no underlying respiratory disease.

**Methods:**

Transcriptional profiling experiments were performed using the Agilent Whole Human Genome 4X44K array platform. Time point-based microarray and principal component analyses were conducted to identify and distinguish acute respiratory illness-associated transcriptional profiles over the course of our study. Gene enrichment analysis was conducted to identify biological processes over-represented within each acute respiratory illness-associated profile, and gene expression was subsequently confirmed by quantitative polymerase chain reaction.

**Results:**

We found that acute respiratory illness is characterized by dynamic, time-specific transcriptional profiles whose magnitudes of expression are influenced by underlying respiratory disease and the mucosal repair signature evoked during acute respiratory illness. Most strikingly, we report that people with asthma who experience acute respiratory illness-induced exacerbations are characterized by a reduced but prolonged inflammatory immune response, inadequate activation of mucosal repair, and the expression of a newly described exacerbation-specific transcriptional signature.

**Conclusion:**

Findings from our study represent a significant contribution towards clarifying the complex molecular interactions that typify acute respiratory illness-induced asthma exacerbations.

## Background

Acute respiratory illness (ARI) is a leading cause of morbidity across all demographic and socioeconomic strata worldwide and the primary trigger for exacerbations of chronic lower airway diseases including asthma [1,2]. While the genes and expression patterns characterizing the peak of ARI symptoms are well described [3-6], understanding of the molecular events during the resolution of ARI remains limited. Moreover, it remains to be determined how the presence of underlying allergic respiratory diseases such as asthma influence molecular events over the course of ARI (that is, peak to resolution of symptoms).

Transcriptional profiling studies have revealed that the pathophysiology, phenotypes and therapeutic response of asthma are accompanied by distinct gene expression profiles [7-14]. However, despite accounting for the highest proportion of all asthma-related health-care expenditures [15,16], little is known of the molecular events that characterize ARI or other environmentally induced (for example, pollen, dander, chemicals) episodes of exacerbations.

Comparative profiling studies between exacerbation and a ‘stable’ asthmatic baseline have revealed that asthma exacerbations involve complex interactions between acute and chronic and/or allergic states of inflammation [17-20]. However, contemporary profiling studies of exacerbation often exclude non-asthmatic or allergic controls, negating many of the genes and expression patterns identified, particularly in episodes of ARI-induced exacerbation as genuine exacerbation-specific ‘signatures’. Furthermore, despite constituting the initial barrier to exacerbation-inducing exposures [21], few profiling studies have focused on the airway mucosa during exacerbation [22], hindering a comprehensive understanding of the molecular events that precipitate asthma exacerbation.

To address the deficiencies in our understanding of the molecular events that characterize ARI and ARI-induced asthma exacerbations, we undertook a transcriptional profiling study of the nasal mucosa over the course of ARI in individuals with asthma, allergic rhinitis and no underlying respiratory disease. We report that ARI is characterized by dynamic, time-specific transcriptional profiles whose magnitudes of expression are influenced by underlying respiratory disease and the mucosal repair signature evoked during ARI. Most strikingly, people with asthma who experience ARI-induced exacerbations are characterized by a reduced but prolonged inflammatory immune response, inadequate activation of mucosal repair, and the expression of a newly described exacerbation-specific transcriptional signature.

## Methods

### Ethics statement

This report encompassed samples from the Naturally Acquired Upper Respiratory Illness study conducted at University of California, San Francisco (UCSF) from November 2001 to November 2004. The study design was approved by the UCSF Committee of Human Research. This study conformed to the Helsinki Declaration. Written informed consent was obtained for all participants prior to inclusion in the study.

### Study design

Participants with a history of asthma, hay fever or no underlying airway disease were recruited for the study via community advertisements. Upon the onset of ARI or ‘common cold’ symptoms, recruited participants were instructed to attend the first of three required visits to the study clinic. The first and second visits were designed to obtain samples and clinical data during the peak and reduction/resolution stages of symptomatic illness, whereas the third visit would occur when volunteers were asymptomatic and serve as a prospective baseline (BL) for the study. The study population represented attended the first, second and third clinic visits on average 2 days (D2; range 0 to 5 days), 6 days (D6; range 4 to 8 days) and 89 days (range 29 to 297 days) after the onset of ARI symptoms.

At each visit, we obtained nasal scrapings (Rhino-Probe™, Arlington Scientific, Inc., Springville, UT, USA) from the lower turbinates (right side on visit one, left at visit two, both sides at baseline visit) after two nasal lavages (10 mL saline each) to remove overlying mucus. We also measured at each visit exhaled nitric oxide (eNO) and forced expiratory flow in 1 second (FEV1) by performing spirometry. At the BL visit, participants underwent a methacholine test to assess airway hyper-reactivity and an allergy skin test to determine if they were atopic [23]. Participants were instructed to complete diaries to record severity of common cold and chest (asthma) symptoms and peak expiratory flow rate (PEF, using a MiniWright meter, KW-Med Inc., USA) twice a day as previously described [24]. Virus identification in nasal lavage from visit one was performed using PCR-based techniques as previously outlined [25]. We used Friedman repeated-measures rank test to analyze changes across all three time points and Mann Whitney *U*-test for comparisons between groups. Correlations were analyzed using Spearman’s rank correlation.

Asthma exacerbation during ARI was defined *a priori* as either:

1. an increase in asthma symptoms that made the participant increase or start inhaled or oral corticosteroids, or seek medical care in a doctor’s office, urgent care clinic or emergency department or

2. an increase in chest or asthma symptom score by ≥10 points for ≥2 days over average daily scores during baseline week plus at least one of these objective changes: use of ≥4 puffs of albuterol above the average daily use in the baseline week for ≥2 days; decline of ≥10% in FEV1 during ARI (first or second visit) compared to FEV1 at BL; or decline of ≥20% in PEF for ≥2 days during the cold week compared with best PEF achieved in the BL week.

### DNA microarray experiments

Total RNA was extracted from nasal mucosa samples using RNeasy Mini Kit (Qiagen, Valencia, CA, USA) with the addition of a DNAse treatment (Promega, Madison, WI, USA) and re-purification phase. Extracted RNA was further purified using the RNA Clean and Concentrator (ZYMO Research, Irvine, CA, USA) and quality assessed by RNA 6000 Pico Kit and 2100 Bioanalyzer (Agilent Technologies, Santa Clara, CA, USA). From those samples exhibiting high RNA integrity, 650 ng of RNA was submitted to the UCSF Shared Microarray Core Facilities for microarray experiments employing the Whole Human Genome 4X44K array platform (#G4112F, Agilent Technologies). Microarray data has been deposited in the Gene Expression Omnibus [GEO:GSE46171].

### DNA microarray data analysis

Because small group numbers limited the statistical power of group-based analyses, time point-based comparative analyses were conducted using log_2_ normalized gene expression data to identify overall temporal transcriptional profiles of ARI (for example, D2 versus BL). A 4×3 analysis of variance linear model was fitted to the comparisons to estimate the mean expression values and differentially expressed genes identified by calculation of moderated t-statistic, B statistic, false discovery rate and raw *P*-value for each comparison of interest. In addition, a mixed linear model was fitted for each gene to estimate the within-subject correlation. A single robust average of this correlation was used as the within-subject correlation when fitting the linear model for all genes. Adjusted *P*-values were produced by the method proposed by Holm [26]. To encompass the complete range of gene expression driven by ARI in subsequent analyses, no fold-change cutoff was applied to the significant differentially expressed genes identified (all adjusted *P* <0.05). All procedures were carried out using functions in the R package *limma* in *Bioconductor*[27].

### Principal component analysis

Principal component analysis (PCA) was used to identify the primary source of variation driving the differentially expressed genes identified in time point-based microarray analysis (n = 2,456; all adjusted *P* <0.05). All PCAs were performed on covariance matrices of gene expression data represented as log_2_ normalized probe intensities (n = 2,456 gene probes/sample) using PAST (v2.15) [28].

Initial PCA included all matched time point microarray samples from each study group to encompass all potential sources of variation over the course of our study (for example, ARI, disease status, sex and medications). Additional group-based PCAs encompassing three time point transitions (BL to D2, D2 to D6, and D6 to BL) were then conducted using all available microarray samples for each group. To statistically confirm the influence of disease status on the ARI-induced changes in gene expression found by PCA, the separation of time point clusters along the principal component 1 (PC1) axis was determined by analysis of PC1 scores between samples by paired *T*-test (matched samples) or Mann–Whitney *U*-test (unmatched samples).

### Identification of group-based acute respiratory illness-associated transcriptional profiles

Since we regarded PC1 to represent the ARI-associated variation within our dataset, we determined ARI-associated transcriptional profiles by ranking the n = 2,456 genes based on their PC1 loadings. Gene rankings were generated for each group-based BL to D2, D2 to D6, and D6 to BL PCA and were used to identify the ARI-associated profiles that characterized the peak, reduction and resolution stages of each group respectively.

Since no consistent component loading cutoff could be found within the literature, the top n = 100 most positive or negative loading genes were then selected from each group and subjected to comparative analyses (15 in total) using VENNY [29]. For the time points exhibiting a positive PC1 score (that is, D2 of peak and reduction and D6 of resolution), the n = 100 most positive genes (that is, the highest) from each group were compared. For the time points exhibiting a negative PC1 score (that is, D6 of reduction), the n = 100 most negative genes (that is, the lowest) from each group were compared.

Kinetics of gene expression were determined by plotting gene probe intensities at each time point. Probe intensities (log_2_) were scaled so that the maximum intensity of each gene across time points and study groups was 1 and the minimum was 0. Hierarchical cluster analysis of microarray data (log_2_ fold change in expression) was performed with Euclidean distance and average linkage using MEV [30]. Correlation analysis between gene expression and PC1 loadings was conducted to determine how the expression level of the core, shared or distinct genes defined each stage of ARI in each of our study groups. All correlation analyses were performed using Prism 5 for windows (GraphPad Software Inc., La Jolla, CA, USA).

### Gene enrichment analysis

Gene enrichment analysis (GEA) was performed using both MetaCore™ (GeneGo™, Thomson Reuters) and Gene Enrichment Profiler [31]. MetaCore™ identifies enriched biological process and pathways within gene sets using the Gene Ontology™ and GeneGo™ Ontologies, the latter derived from curated databases of experimentally validated interactions. MetaCore™ employs a hypergeometric model to determine significance of functional relationships. Gene Enrichment Profiler generates enrichment analysis of gene sets in various cell types to provide a cell/tissue perspective of large gene expression datasets.

### Quantitative real-time PCR

Total RNA from participants with three (that is, BL, D2 and D6) or two (that is, either combination of BL, D2 or D6) matched samples were selected for quantitative real-time PCR analysis. Missing samples were due to either participant failing to attend one of the three clinic visits or sample RNA quantity being minimal. Genes were selected for expression confirmation based on PC1 loadings. Depending on the stage of ARI analyzed, the highest ranked (peak, D2 of reduction and resolution) or lowest (D6 of reduction) genes were selected. Gene expression in each sample was normalized to glyceraldehyde-3-phosphate dehydrogenase and peptidylprolyl isomerase A as outlined previously [32]. Significance of fold change in gene copy number between groups was determined by Mann Whitney *U*-test. Primer and probe sequences are available upon request.

## Results

### Study design and population characteristics

**Figure 1:**
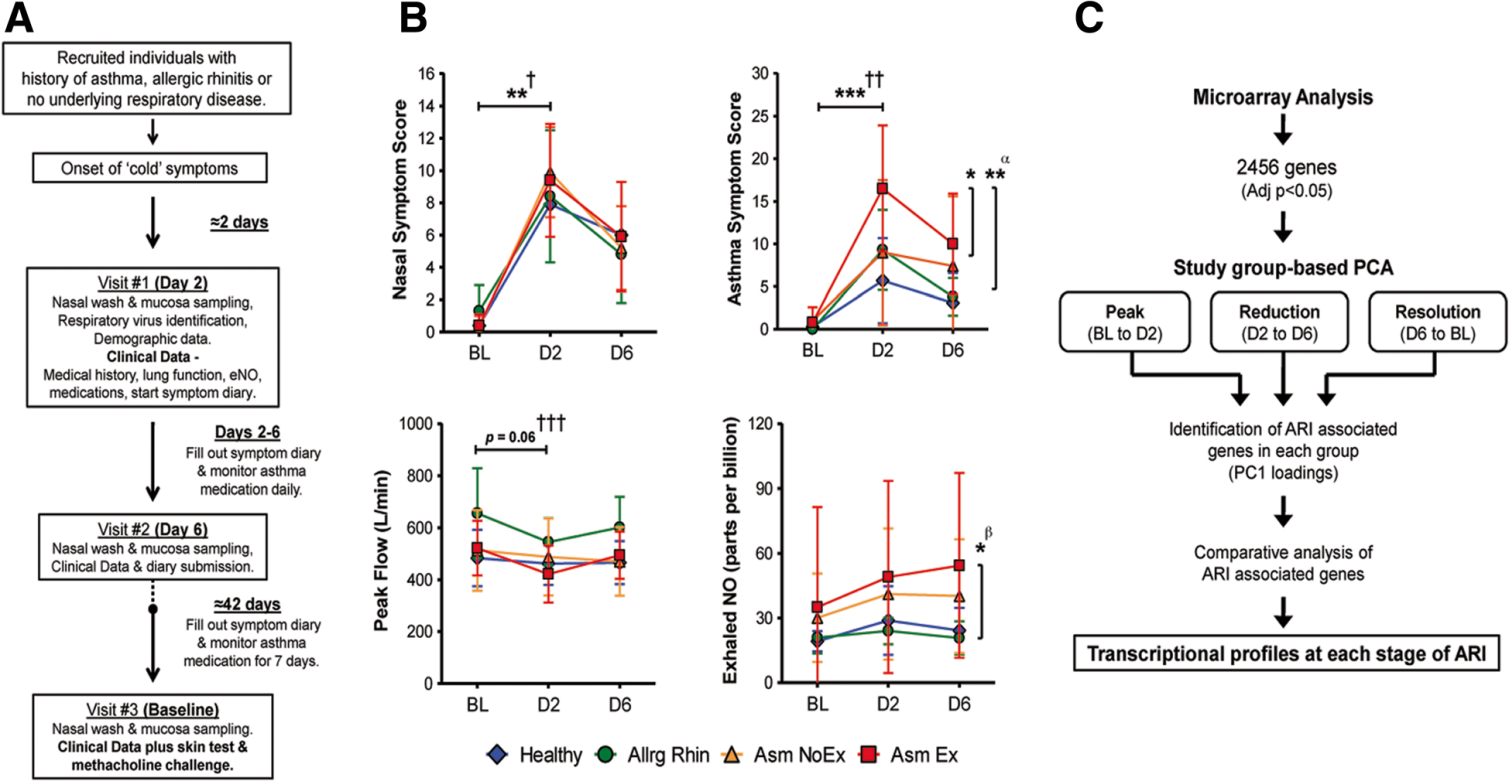
**Study design, clinical characteristics and methods summary. (A)** Naturally acquired upper respiratory infection study design. **(B)** Select clinical characteristics measured during the study period by group. All points depict mean ± SD. **P ≤0.01, ***P ≤0.001. ^†^All groups, ^††^Allrg Rhin, Asm NoEx and Asm Ex only, ^†††^Asm Ex only, ^α^Asm Ex versus Healthy and Asm NoEx at D2, ^β^Asm Ex versus Healthy and Allrg Rhin at D6. Demographic data of study population is outlined in Table 1. **(C)** Summary of the methods employed to determine the transcriptional profiles characterizing the peak, reduction and resolution of acute respiratory illness. ARI, acute respiratory disease; BL, baseline, D2/6, day 2/6; NO, nitric oxide; PCA, principle component analysis.

A summary of the study design is depicted in Figure 1A. Briefly, adults with asthma, allergic rhinitis or no underlying respiratory disease were recruited; after the onset of ‘common cold’ or ARI symptoms they visited the study clinic for nasal mucosa sampling on D2 and D6 of symptomatic illness, and an asymptomatic BL sample was taken at least 29 days later. Based on study data, clinical history, allergy skin test and methacholine challenge, participants were categorized into one of four study groups: Healthy (that is, no underlying respiratory disease), Allergic Rhinitis (Allrg Rhin), Asthmatics without an episode of exacerbation (Asm NoEx), and Asthmatics with an episode of exacerbation during the study period (Asm Ex; Figure S1A in Additional file 1). Each study group experienced a significant increase in nasal symptoms between BL and D2. However, the most severe asthma symptoms, airway obstruction (as measured by peak flow) and highest levels of eNO, were observed within the Asm Ex group (Figure 1B). Respiratory viruses were detected at a similar rate among all groups (Table 1).

**Table 1:** Demographic and clinical data of the study population

	**Healthy**	**Allrg Rhin**	**Asm NoEx**	**Asm Ex**	**Total**
**Participants, n (%)**	9 (18.0)	7 (14.0)	23 (46.0)	11 (22.0)^a^	50 (100.0)
**Female, n (%)**	7 (14.0)	3 (6.0)	18 (36.0)	10 (20.0)	38 (76.8)
**Age; median years (max-min)**	30 (18 to 37)	31 (20 to 47)	37 (19 to 66)	29 (25 to 46)	32 (18 to 66)
**Ethnicity, n (%)**					
White	8 (16.0)	5 (10.0)	13 (26.0)	9 (18.0)	35 (70.0)
African American	0 (0.0)	1 (2.0)	4 (8.0)	1 (2.0)	6 (12.0)
Hispanic	0 (0.0)	0 (0.0)	3 (6.0)	0 (0.0)	3 (6.0)
Other	1 (2.0)	1 (2.0)	3 (6.0)	1 (2.0)	6 (12.0)
**Atopic, n (%)**	1 (2.0)	7 (14.0)	18 (36.0)	8 (16.0)	34 (68.0)
**Baseline PC**_**20**_**; mean mg/mL (±SD)**	28 (27.1)	6.7 (3.8)	4.5 (5.1)	4.4 (5.2)	n/a
**Baseline FEV1; mean% predicted (±SD)**	98.3 (11.0)	105.8 (6.7)	97.8 (16.5)	91.2 (10.8)	n/a
**Medication, n (%)**					
Inhaled corticosteroids	0 (0.0)	0 (0.0)	6 (12.0)	3 (6.0)	9 (18.0)
Inhaled corticosteroids plus long-acting beta agonists	0 (0.0)	0 (0.0)	2 (4.0)	1 (2.0)	3 (6.0)
Nasal steroids	0 (0.0)	0 (0.0)	6 (12.0)	3 (6.0)	9 (18.0)
**Respiratory virus identification, n (%)**					
Human rhinovirus	5 (10.0)	1 (2.0)	9 (18.0)	3 (6.0)	18 (36.0)
Respiratory syncytial virus	0 (0.0)	0 (0.0)	2 (4.0)	1 (2.0)^b^	3 (6.0)
Human coronavirus	1 (2.0)	1 (2.0)	2 (4.0)	2 (4.0)^b^	6 (12.0)
Influenza A virus	0 (0.0)	1 (2.0)	1 (2.0)	0 (0.0)	2 (4.0)
Human parainfluenza virus	0 (0.0)	1 (2.0)	0 (0.0)	0 (0.0)	1 (2.0)
No detection	3 (6.0)	3 (6.0)	9 (18.0)	5 (10.0)	20 (40.0)

All bracketed values represent percentage of total participants unless otherwise stated. ^a^Among the Asm Ex group, n = 4 were classified because of increase/start of corticosteroid or need for medical care and n = 7 were classified due to increased symptoms accompanied by either an increase in albuterol (n = 5 out of 7) or a decline in FEV1 (n = 3 out 7) or peak expiratory flow (n = 6 out of 7). ^b^Includes dual detections. FEV1, forced expiratory volume in 1 s; PC_20_, provocative concentration of methacholine causing a 20% fall in FEV1; SD, standard deviation.

### Identification of acute respiratory illness-associated transcriptional profiles

Given that all study groups experienced the complete course of ARI symptoms (Figure 1B), we sought initially to identify the temporal transcriptional profile of ARI (Figure 1C). Employing all microarray samples (Figure S1B), time-point based microarray analysis (that is, D2 versus BL, D6 versus D2 and D6 versus BL) revealed a total of 2,456 genes exhibiting differential expression over the course of our study (all adjusted *P* <0.05; data not shown). PCA confirmed that the primary source of variation driving the differential expression (that is, principal component 1 - PC1) was time-dependent (Figure S2 in Additional file 1). Considering ARI would be the only time-dependent biological phenomenon consistent across all study groups, we reasoned that the genes that defined PC1 the most (as measured by component loadings) would therefore represent the transcriptional profile of ARI.

Group-based PCA then indicated that the differential gene expression characterizing the transition between study time points was influenced by disease status (Figure S3A in Additional file 1). To investigate when underlying disease influenced the transcriptional profiles of ARI, we conducted additional group-based PCAs encompassing the peak (BL to D2), reduction (D2 to D6) and resolution (D6 to BL) of ARI symptoms (Figure S3B-D in Additional file 1) and compared the ARI-associated profiles of each study group respectively (Figure 1C).

### Peak stage (baseline to day 2)

**Figure 2:**
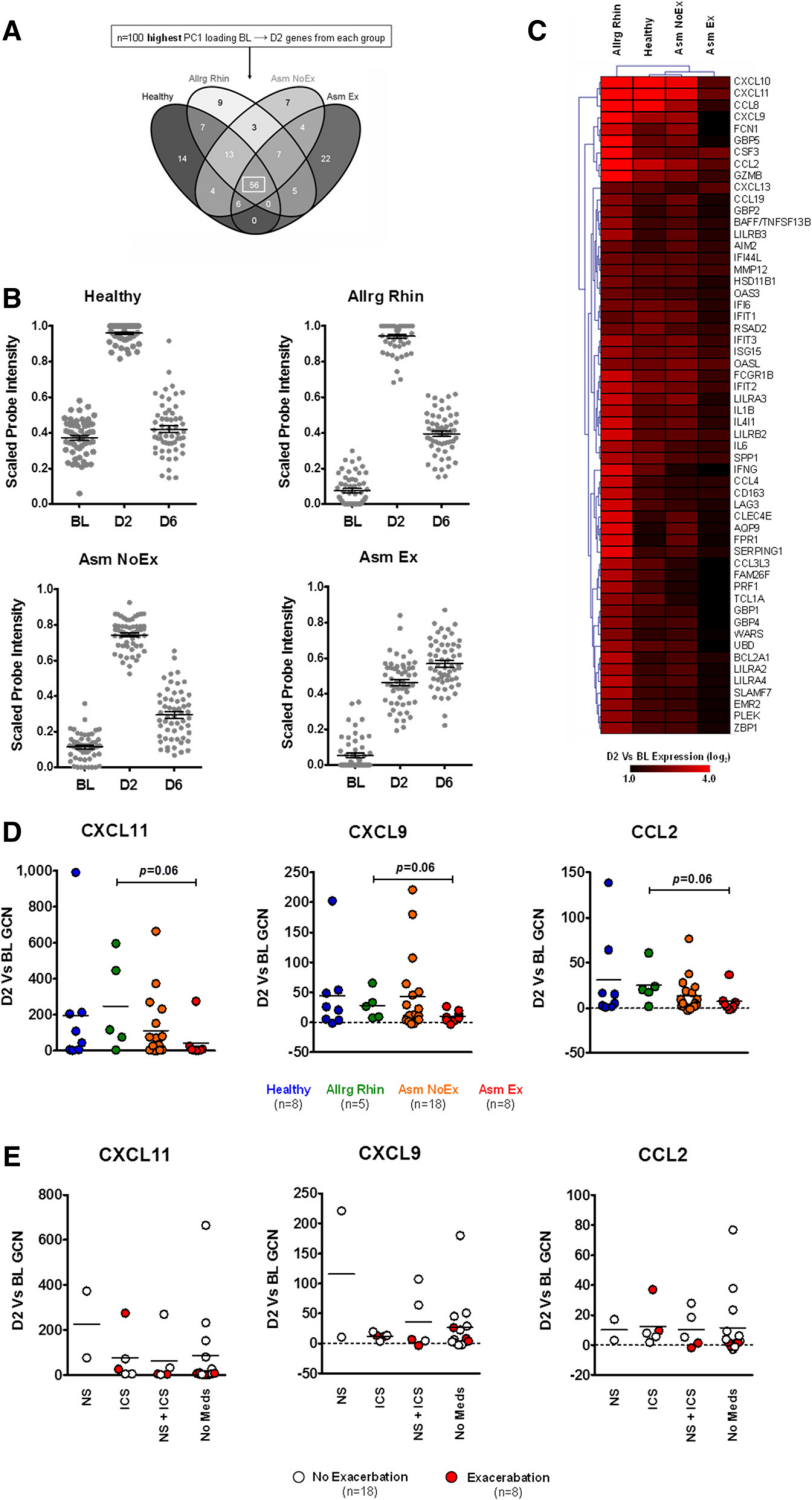
**Peak stage of acute respiratory illness. (A)** Comparative analysis of the acute respiratory illness-associated genes from each group revealed that D2 was defined by a core transcriptional profile regardless of disease status (box). Complete gene lists for each comparison are outlined in Table S1 in Additional file 2. **(B)** Kinetics of core-D2 gene expression in each study group (n = 56 probes/time point). **(C)** Hierarchical clustering analysis of the D2 versus baseline microarray expression of the core-D2 genes. **(D)** Quantitative real-time PCR confirmation of select core-D2 gene expression. Horizontal line depicts mean. Number of matched BL and D2 samples from each group used for quantitative real-time PCR assays are indicated below. **(E)** Influence of medication use on core-D2 gene expression for all asthmatics. Horizontal line depicts mean. BL, baseline; *CXCL11*/*9*, Chemokine (C-X-C motif) ligands; *CCL2,* Chemokine (C-C motif) ligand; D2, day 2; D6, day 6; GCN, fold change in normalized gene copy number; ICS, inhaled corticosteroids; NS, nasal steroids.

Consistent with the dramatic increase in symptoms (Figure 1B), analysis of group-based PCA indicated all study groups exhibited a clear transition between BL and D2 (Figures S3B and S4A in Additional file 1). Since our study concentrated on the influence of underlying respiratory disease during ARI and not at the asymptomatic baseline, we identified and focused on the ARI-associated profiles of the D2 time point for each study group (see Methods). Comparative analysis of ARI-associated profiles revealed 56 common genes, indicating that the peak stage of ARI was characterized by a ‘core’ transcriptional profile that was independent of underlying respiratory disease (Figure 2A and Table S1 in Additional file 2).

GEA determined that the core-D2 genes were associated with proinflammatory/immune processes, pathways and cells (Table S2 in Additional file 3). The transition towards D2 was associated predominantly with the presence of respiratory virus, as shown by an analysis of virus identification (Figure S4B in Additional file 1), and microarray expression data showed a robust increase in core-D2 gene expression (Figure 2B). These results indicate that the transcriptional profile of the peak stage reflects activation of the immune response.

Although the positive expression of the core-D2 genes defined the peak stage in all study groups, further investigation indicated that the presence of asthma exacerbation influenced the magnitude of core-D2 gene expression (Figure 2C and Figure S5A in Additional file 1). Quantitative real-time PCR analysis of select core-D2 gene expression in microarray and additional study samples (Figure S1 in Additional file 1) subsequently confirmed that at the peak stage of ARI, asthma with exacerbation was characterized by an expression pattern consistent with reduced activation of the inflammatory immune response that occurred regardless of the presence or absence of medications (Figure 2D,E).

### Reduction stage (day 2 to day 6)

While nasal symptoms declined between D2 and D6 in all study groups, the Asm Ex group maintained heightened asthma symptoms and eNO levels (Figure 1B). Analysis of group-based PCA subsequently revealed that the presence of asthma exacerbation specifically influenced the transition between D2 and D6 (Figures S3C and S4C in Additional file 1). To identify which time point and corresponding ARI-associated profile was affected the most by exacerbation, we investigated and compared the ARI-associated profiles for both the D2 and D6 time points.

#### Acute respiratory illness-associated profiles of day 2

**Figure 3:**
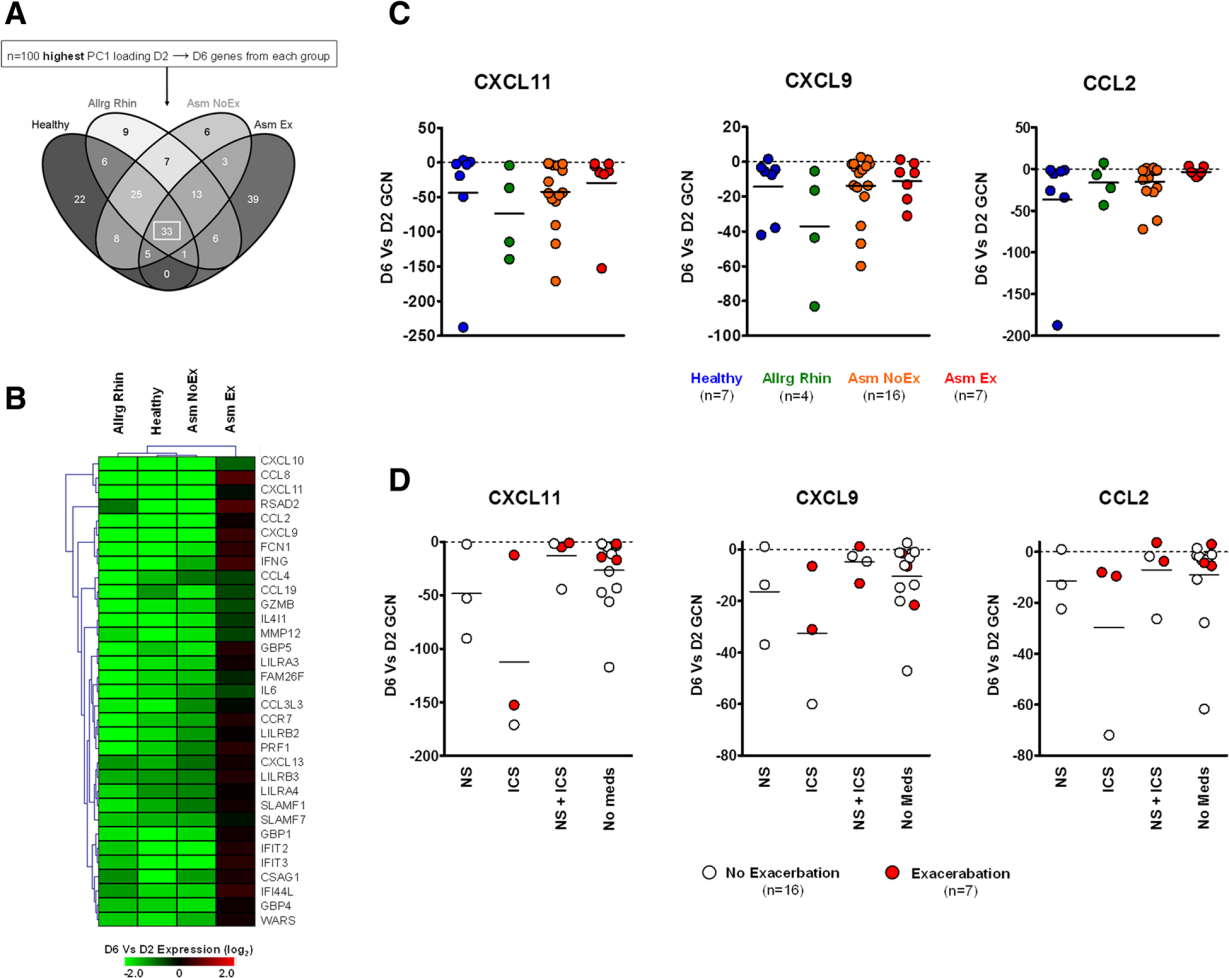
**Day 2 at the reduction stage of acute respiratory illness. (A)** Comparative analysis of the acute respiratory illness-associated genes of D2 with respect to D6 indicated a core of n = 33 genes across all study groups (box). Complete gene lists generated for each comparison are outlined in Table S1 in Additional file 2. **(B)** Hierarchical clustering analysis of the D6 versus D2 microarray expression of the core-D2 genes. **(C)** Quantitative real-time PCR confirmation of select core-D2 gene expression. Horizontal line depicts mean. Number of matched D2 and D6 samples from each group used for quantitative real-time PCR assays are indicated below. **(D)** Influence of medication use on core-D2 gene expression for all asthmatics. Horizontal line depicts mean. *CXCL11*/*9*, Chemokine (C-X-C motif) ligands; *CCL2,* Chemokine (C-C motif) ligand; D2, day 2; D6, day 6; GCN, fold change in normalized gene copy number; ICS, inhaled corticosteroids; NS, nasal steroids.

Comparative analysis of the ARI-associated D2 profiles (with respect to D6) indicated that 33 genes were common (Figure 3A and Table S1 in Additional file 2). Subsequent comparative analysis revealed that 30 of these 33 genes were shared with core-D2 genes previously identified at the peak stage (data not shown). However, while a vigorous negative expression pattern defined the D2 time point amongst the Healthy, Allrg Rhin and Asm NoEx groups, the Asm Ex group exhibited prolonged positive expression of the core-D2 genes (Figure 3B and Figure S5B in Additional file 1). Quantitative real-time PCR analysis of select core-D2 gene expression indicated that at the reduction stage of ARI, asthmatics with exacerbation were characterized by an expression pattern consistent with inadequate modulation of the inflammatory response that occurred in the presence and absence of medications (Figure 3D,E).

#### Acute respiratory illness-associated profiles of day 6

**Figure 4:**
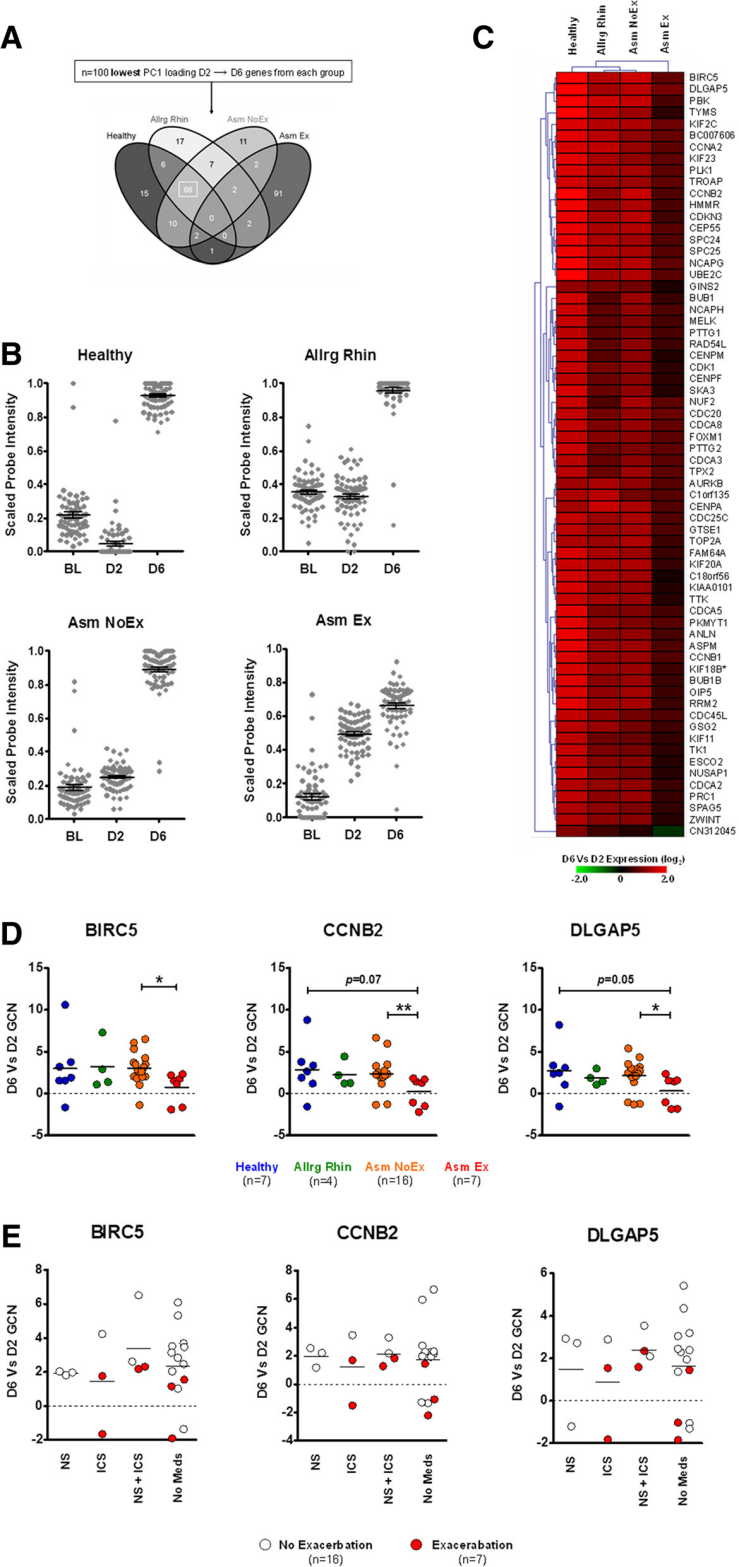
**Day 6 at the reduction stage of acute respiratory illness. (A)** Comparative analysis of acute respiratory illness-associated genes of D6 indicated that n = 66 were shared amongst the Healthy, Allrg Rhin and Asm NoEx groups (box). Complete gene lists generated for each comparison are outlined in Table S1 in Additional file 2. **(B)** Kinetics of shared-D6 gene expression in each study group (n = 66 probes/time point). **(C)** Hierarchical clustering analysis of the D6 versus D2 microarray expression of the shared-D6 genes. **(D)** Quantitative real-time PCR confirmation of select shared-D6 gene expression. Horizontal line depicts mean. *P = <0.05, **P ≤0.01. Number of matched D2 and D6 samples from each group used for quantitative real-time PCR assays are indicated below. **(E)** Influence of medication use on shared-D6 gene expression for all asthmatics. Horizontal line depicts mean. *BIRC5*, Baculoviral IAP repeat containing 5; *CCNB2*, Cyclin B2; *DLGAP5*, Discs, large (Drosophila) homolog-associated protein 5; GCN, fold change in normalized gene copy number; D2, day 2; D6, day 6; ICS, inhaled corticosteroids; NS, nasal steroids.

Comparative analysis of the ARI-associated D6 profiles revealed no common genes existed across study groups. Rather, 66 genes were shared amongst the Healthy, Allrg Rhin and Asm NoEx groups and the Asm Ex group was characterized almost exclusively by a set of 91 distinct genes (Figure 4A and Table S1 in Additional file 2).

We focused initially on the 66 ARI-associated genes shared between the Healthy, Allrg Rhin and Asm NoEx groups. Using GEA, we determined that these shared-D6 genes were associated with processes and pathways consistent with mucosal repair and recruitment of immune cells (Table S2 in Additional file 3). However, while the robust expression of repair-associated genes defined the D6 time point amongst the Healthy, Allrg Rhin and Asm NoEx groups, the Asm Ex group exhibited an earlier and reduced expression pattern (Figure 4B and Figure S5C in Additional file 1). Quantitative real-time PCR analysis subsequently confirmed that at the reduction stage of ARI, asthmatics with exacerbation were characterized by an expression pattern consistent with inadequate mucosal repair in the presence and absence of medications (Figure 4D,E).

**Figure 5:**
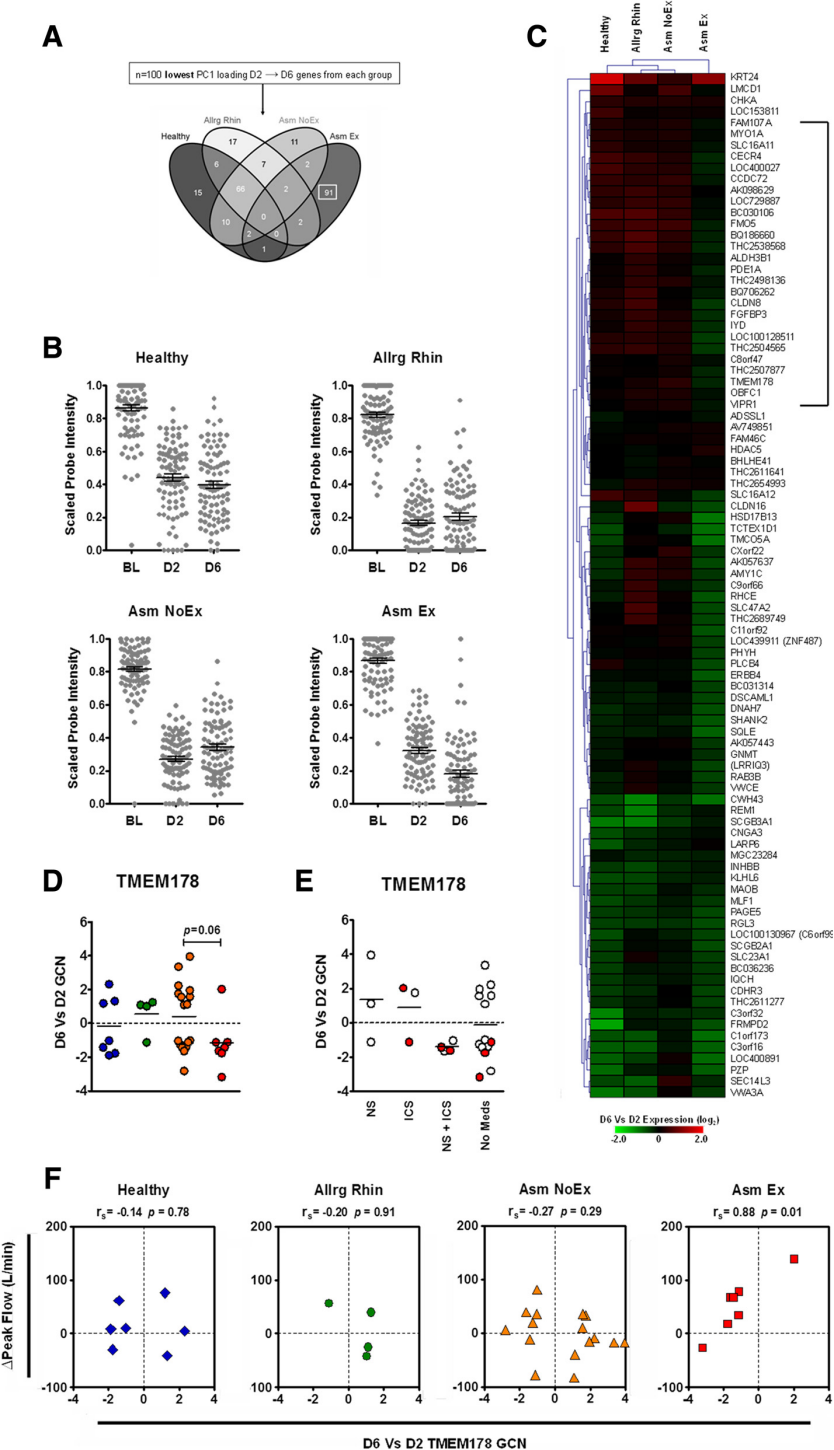
**Analysis of the genes characterizing the asthma with exacerbation group (Asm Ex) during the reduction stage of acute respiratory illness. (A)** Comparative analysis of acute respiratory illness-associated genes of D6 revealed the Asm Ex group was defined almost exclusively by a distinct set of genes (box). **(B)** Kinetics of shared-D6 gene expression in each study group (n = 91/time point). **(C)** Hierarchical clustering analysis of the D6 versus D2 microarray expression of the distinct Asm Ex genes revealing a subset exhibited an exacerbation-specific negative expression pattern (n = 27; bracketed). **(D)** Quantitative real-time PCR confirmation of *TMEM178* gene expression. Sample numbers for each group are depicted in Figure 4D. **(E)** Influence of medication use on *TMEM178* gene expression for all asthmatics. Horizontal line depicts mean. **(F)** Relationship between changes in *TMEM178* gene expression and airway obstruction for each study group. A significant correlation was only observed for the Asm Ex group. BL, baseline; D2, day 2; D6, day 6; GCN, fold change in normalized gene copy number; ICS, inhaled corticosteroids; NS, nasal steroids; r_s_, Spearmans rho; TMEM, transmembrane protein.

We then focused on the 91 ARI-associated D6 genes of the Asm Ex group (Figure 5A). Using GEA, we determined that only a very limited number of these distinct-D6 genes were associated with known biological processes, pathways or cell types (Table S2 in Additional file 3). Kinetics of distinct-D6 genes indicated an overall reduced expression within the Asm Ex group at D6 (Figure 5B). Further investigation revealed that a subset of the distinct-D6 genes exhibited an Asm Ex-specific pattern of expression (Figure 5C and Figure S5D in Additional file 1). Quantitative real-time PCR analysis confirmed that the exacerbation-specific expression of transmembrane protein (TMEM) 178 occurred regardless of the presence or absence of medications (Figure 5D,E). Strikingly, changes in *TMEM178* gene expression were found to be associated with lower airway obstruction exclusively within the Asm Ex group (Figure 5F). Taken together, these results indicate that in addition to inadequate mucosal repair at the reduction stage of ARI, asthma with exacerbation was characterized by an exacerbation-specific profile and expression pattern that could be associated with a clinical manifestation of exacerbation.

### Resolution stage (day 6 to baseline)

**Figure 6:**
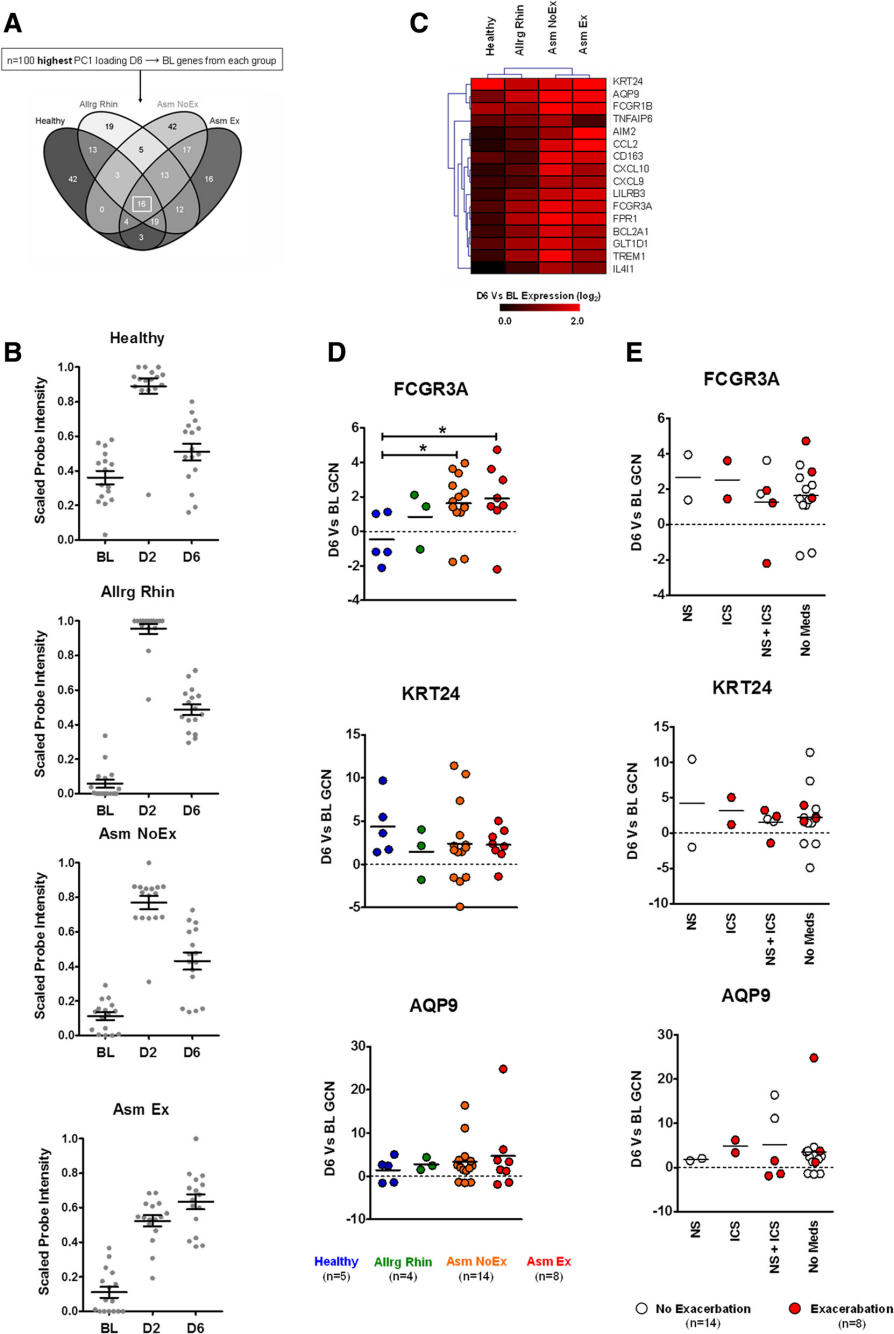
**Resolution stage of acute respiratory illness. (A)** Comparative analysis of acute respiratory illness-associated genes of D6 with respect to BL revealed that the resolution stage was characterized by a core transcriptional profile regardless of disease status (box). Complete gene lists generated for each comparison are outlined in Table S1 in Additional file 2. **(B)** Kinetics of shared-D6 gene expression in each study group (n = 16/time point) **(C)** Hierarchical clustering analysis of the D6 versus BL microarray expression of the core-D6 genes. **(D)** Quantitative real-time PCR confirmation of select core-D6 gene expression. Horizontal line depicts mean. *P ≤0.05. **(E)** Influence of medication use on core-D6 gene expression for all asthmatics. Horizontal line depicts mean. *AQP9*, Aquaporin 9; BL, baseline; GCN, fold change in normalized gene copy number; D2, day 2; D6, day 6; *FCGR3A*, Fc fragment of immunoglobulin G, low affinity IIIa, receptor (CD16a); *KRT24*, Keratin 24.

Consistent with decline in symptoms (Figure 1B), group-based PCA indicated that all groups exhibited a clear transition between D6 and BL (Figures S3D and S4D in Additional file 1). Comparative analysis of the ARI-associated D6 genes (with respect to BL) revealed that 16 were common (Figure 6A and Table S1 in Additional file 2). Subsequent comparative analysis indicated 11 of the 16 D6 genes were shared with the core-D2 genes previously identified at the peak stage (Figure 2A), indicating that in relation to BL, the D6 time point exhibited more of an inflammatory-associated profile.

Kinetic analysis revealed that the Asm Ex group maintained the highest expression of core-D6 genes (Figure 6B,C). Remarkably, however, further investigation revealed that the D6 time point amongst the Healthy group was defined by a limited expression pattern of the core-D6 genes (Figure 6D and Figure S5E in Additional file 1). Quantitative real-time PCR analysis of select core-D6 genes confirmed that the Healthy group exhibited the lowest expression level of immune-associated genes (encoding Fc fragment of immunoglobulin G, low affinity IIIa, Aquaporin 9) and the heightened expression of cell structure-associated genes (encoding Keratin 24, Figure 6E). Taken together, these results indicate that at the resolution stage of ARI, the absence of underlying respiratory disease results in a quicker resolution of inflammation and continued activation of mucosal repair.

## Discussion

**Figure 7:**
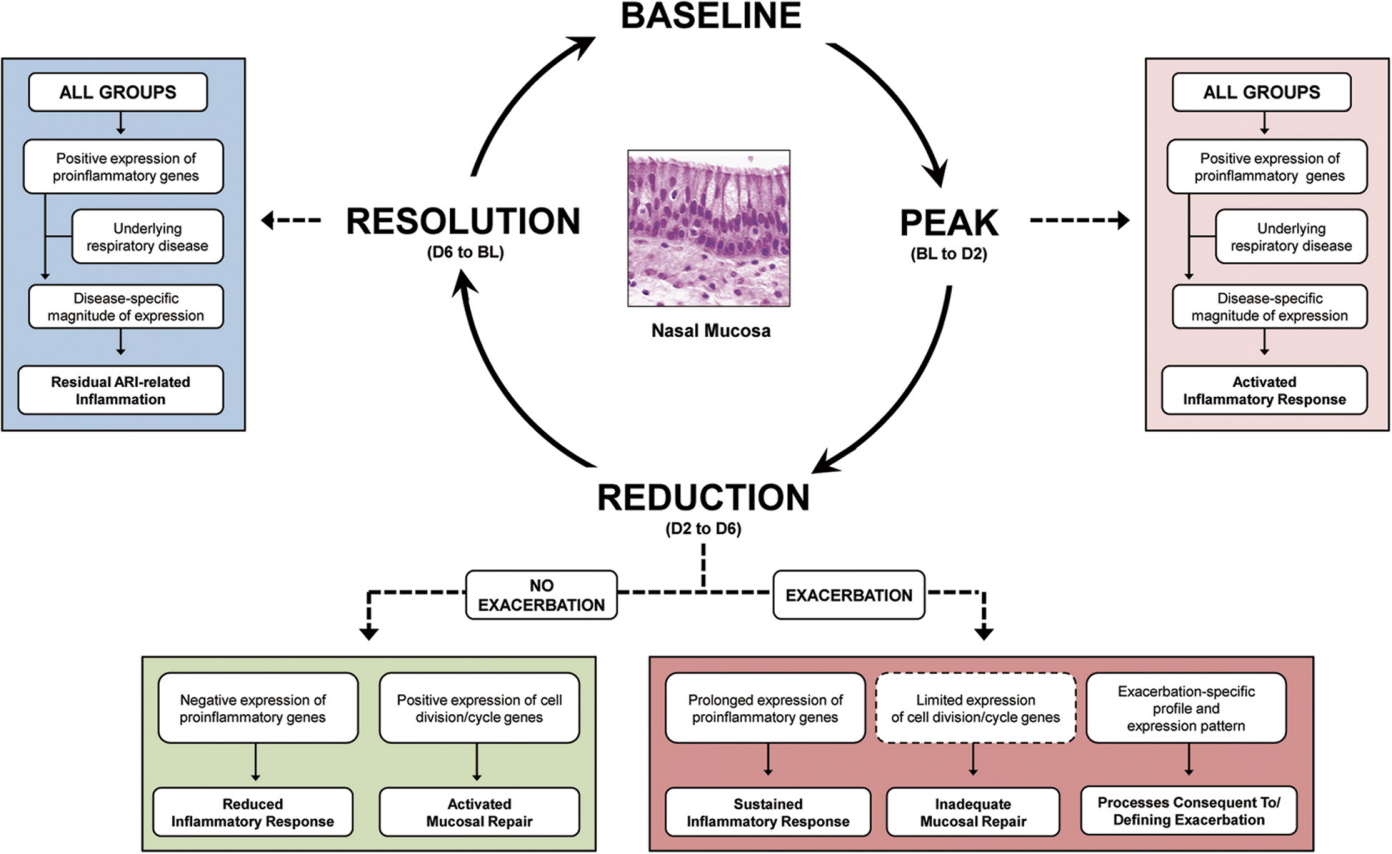
**Proposed stages of acute respiratory illness.** Derived from the analysis of three time point transitions of ARI, our model proposes that ARI can be divided into three stages reflecting the peak, reduction and resolution of symptoms. By encompassing clinically distinct groups, we additionally propose that the presence of underlying respiratory disease, particularly asthma exacerbation, can influence either the magnitude of gene expression or the transcriptional profiles that characterize the distinct biological processes at each respective stage of ARI. ARI, acute respiratory illness; BL, baseline; D2, day 2; D, day 6.

To address the deficiencies in our understanding of the mechanisms characterizing ARI and ARI-induced asthma exacerbations, we undertook a transcriptional profiling study of the nasal mucosa over the course of ARI amongst asthmatics, allergic non-asthmatics and otherwise healthy controls. Employing a systems biology approach, we have identified for the first time the transcriptional profiles and corresponding biological processes that characterize the scope of ARI symptoms amongst four clinically distinct groups. From the results obtained, we propose that ARI can be categorized into three stages and that underlying respiratory disease can influence the transcriptional profiles and/or expression patterns that define each stage of ARI (Figure 7).

The peak stage of ARI is defined by the robust expression of proinflammatory-associated genes and coincides with the greatest severity of ARI symptoms (Figure 7). Consistent with previous studies of respiratory virus infection [4-6], the clinical presentation, transcriptional profiles and expression patterns identified in our study signify the activation of the immune response. However, our study has revealed for the first time that the height of ARI symptoms is characterized by the expression of a core set of genes regardless of the presence of underlying respiratory disease (Figure 2C).

The identification of a core transcriptional profile during the peak stage of ARI is to be expected since ARI is frequently viral in origin and the immune response of the nasal mucosa is predominantly innate [33]. In addition, the ‘common cold’ presentation experienced by all study participants (Figure 1B) would also suggest that the molecular events orchestrating ARI are conserved and therefore impartial to the presence of underlying respiratory disease. Remarkably, however, we observed that clinically distinct groups exhibited specific preference for (as measured by PC1 loadings) and expression levels of the core genes (Figures 2C,D and Figure S5 in Additional file 1), indicating that underlying respiratory disease can influence the intensity and nature of the innate immune response of the airway mucosa.

The most prominent finding from the peak stage of ARI was that asthmatics with exacerbation exhibited an expression pattern consistent with reduced activation of the inflammatory immune response (Figure 2D). Although our findings bolster previous reports of deficient innate antiviral and other immune responses in asthmatics, particularly during episodes of exacerbation [20,34-36], the expression patterns identified could have also reflected the use of asthma medications or the absence of detectable virus infection during exacerbation (Table 1 and Figure S4B in Additional file 1). However, we observed little effect of asthma medications on inflammatory gene expression and no reduction in ARI symptoms or eNO levels within the Asm Ex group (Figures 1B and 2E), suggesting that either the presence of inflammatory cell infiltration (Table S2 in Additional file 3) or other non-immune processes within the airway mucosa were driving ARI symptoms and the development of exacerbation.

The reduction stage represents a unique period of ARI where symptoms are present but in decline (Figure 1B). It is this stage in which the difference between asthmatics experiencing an exacerbation and other individuals with ARI is most evident (Figure 7). The reduction stage of ARI is defined by transcriptional profiles and expression patterns corresponding to two distinct biological processes; the modulation of the inflammatory immune response (Figure 3) and the activation of mucosal repair mechanisms (Figure 4).

Although likely intuitive, our study found that the same core inflammatory genes that characterized the peak in symptoms also characterized the reduction in symptoms when exhibiting a negative expression pattern (Figure 3). Importantly, we identified the transcriptional profile that characterizes the repair mechanisms of the nasal mucosa (Figure 4D,E) and found that, in addition to the ability to modulate the inflammatory immune response, the simultaneous (or subsequent) initiation of mucosal repair through cellular division and immune cell recruitment paralleled the reduction of ARI symptoms (Figure 1B). Interestingly, the transcriptional profiles identified in our study were highly similar to those found in lower airway mucosa in response to mechanical injury [37], indicating the same innate repair mechanism likely exists throughout the entire airways. Results from our study also suggest that in addition to targeting select proinflammatory components (for example, CXCL family members), augmentation of repair mechanisms within the nasal mucosa may provide therapeutic relief from ARI symptoms.

A major finding at the reduction stage of ARI was that the presence of asthma exacerbation dramatically influenced how the inflammatory response was modulated and mucosal repair mechanisms initiated. Asthmatics with exacerbation were characterized by sustained asthma symptoms, high levels of eNO (Figure 1B) and a blunted expression pattern of the genes associated with mucosal repair (Figure 4), despite having a similar proportion of medication use to asthmatics with no exacerbations (63% versus 60%; Table 1). In addition to highlighting the ineffectiveness of current exacerbation treatments [38,39], these findings suggest that a deficient repair response similar to that observed *in vitro*[21,40,41] is present within the airway epithelium of asthmatics during exacerbation.

The most striking finding from the reduction stage was that asthmatics with ARI-induced exacerbations were characterized by a distinct transcriptional profile within the nasal mucosa (Figure 5). Remarkably, the majority of the exacerbation-specific genes identified were found to lack any association with known biological processes, pathways or cell types (Table S2 in Additional file 3), further supporting the view that uncharacterized or otherwise neglected mechanisms (that is, non-immune/inflammatory) contribute to the development of ARI-induced exacerbations.

We identified that a subset of the exacerbation genes exhibited an exacerbation-specific expression pattern (Figure 5C). Changes in expression of one of these genes, *TMEM178*, occurred regardless of medication use and was associated with lower airway obstruction (Figure 5E,F). *TMEM16A* is involved in mucus production and airway smooth muscle contraction [42,43], suggesting the expression of TMEM family members contributes to the pathophysiology of asthma. While further characterization of *TMEM178* is required, we consider *TMEM178* and the other exacerbation genes identified within our study to represent a genuine exacerbation signature and potential targets for exacerbation-specific therapies.

The third and final stage of ARI reflects the residual activity of the inflammatory immune response and the return towards an asymptomatic baseline (Figure 7). Defined by the positive expression of a small core of proinflammatory genes, the resolution stage of ARI was exceptional in that the absence of underlying respiratory disease was found to be the most distinctive feature. Findings from our study revealed that otherwise healthy individuals were characterized by an expression pattern consistent with a reduced proinflammatory immune response and continued mucosal repair, reflecting a quicker resolution of ARI in the absence of underlying respiratory disease (Figure 6C-E).

## Conclusion

By determining the transcriptional profiles characterizing the peak to resolution of ARI symptoms amongst asthmatic and allergic non-asthmatic individuals and otherwise healthy controls, we have revealed that the presence of asthma exacerbation has a dramatic influence on the molecular events that characterize ARI. Our study indicates that people with asthma who experienced ARI-induced exacerbation are characterized by a reduced but prolonged inflammatory immune response, inadequate activation of mucosal repair mechanisms and, most strikingly, a distinct set of genes constituting an exacerbation-specific transcriptional signature. Although the absence of corresponding cellular composition data of the nasal mucosa (for example, via flow cytometry) and the focus exclusively on the upper airways may have been limitations of our study, these findings represent a significant contribution towards clarifying the complex molecular interactions that characterize ARI-induced asthma exacerbations.

## Abbreviations

Allrg Rhin: patients with allergic rhinitis; ARI: acute respiratory illness; Asm Ex: patients with asthma and exacerbation; Asm NoEx: patients with asthma without exacerbation; BL: baseline; CCL: Chemokine (C-C motif) ligand; CXCL: Chemokine (C-X-C motif) ligand; D2: day 2; D6: day 6; eNO: exhaled nitric oxide; FEV1: forced expiratory flow in 1 second; GEA: gene enrichment analysis; ICS: inhaled corticosteroids; NS: nasal steroids; PC1: principal component 1; PCA: principal component analysis; PCR: polymerase chain reaction; PEF: peak expiratory flow rate; TMEM: transmembrane protein.

## Competing interests

The authors declare that they have no competing interests.

## Authors’ contributions

PCA and HAB conceived and designed the study. TW was involved in study recruitment and sample collection. SF, JS, PM and AsB processed samples. DJE, AnB, RB and CE conducted the micorarray experiments. SB conducted principal components analysis. PM conducted data analysis and qPCR experiments. PM, SB, PCA and RPS drafted the manuscript. All authors read and approved the final manuscript.

## Supplementary Material

Additional file 1 Contains recruitment and numbers of participants represented in the current study and PCA. Click here for file

Additional file 2 Contains lists of common, shared or distinct genes identified in comparative analysis of ARI-associated profiles for the peak, reduction and resolution stages of ARI. Click here for file

Additional file 3 Contains results of GEA of gene sets. Click here for file
