# Supplementary material for: Asthmatics with exacerbation during acute respiratory illness exhibit unique transcriptional signatures within the nasal mucosa
Source: Genome Med. 2014 Jan 17;6(1):1. doi: 10.1186/gm520 (PMC3971347; doi:10.1186/gm520)
Supplement: Additional file 1 — Contains recruitment and numbers of participants represented in the current study and PCA. [file gm520-S1.pdf]

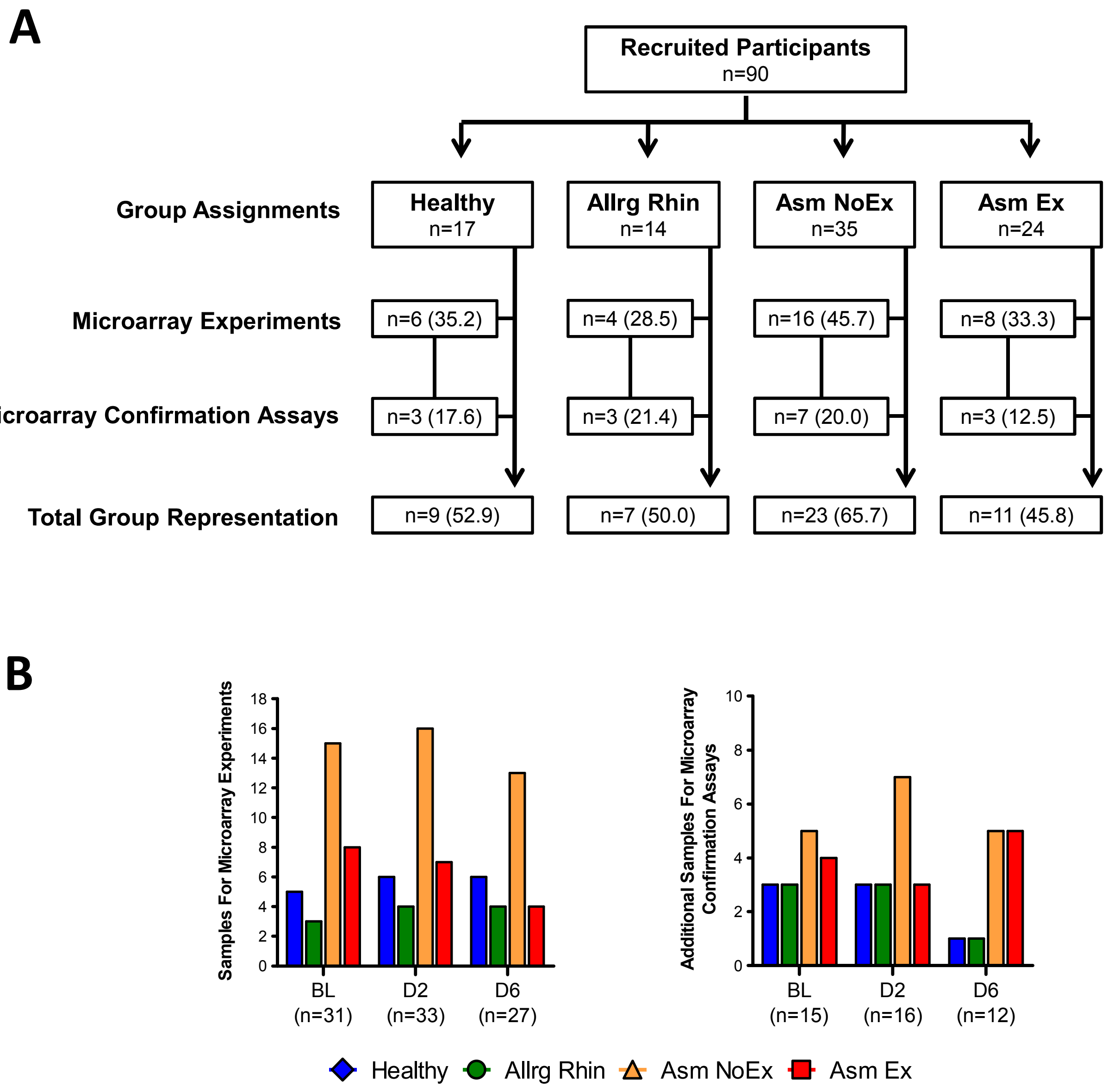

Supplementary Figure S1: Study recruitment and samples distribution. **A.** NATURI recruitment and group representation in current study. Bracketed values represent percentage of total participants represented in current study. **B.** Distribution of nasal mucosa samples used in microarray experiments or gene expression confirmation assays by group and study time point. Total samples per time point are indicated below.

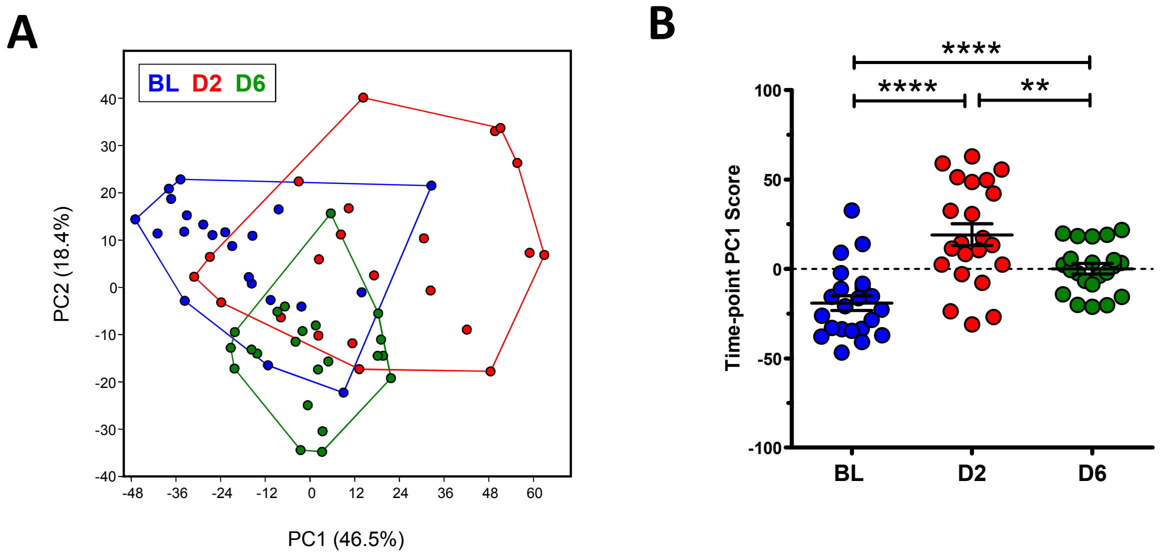

Supplementary Figure S2: Principal component analysis (PCA) of gene expression dataset. **A.** PCA employing all matched BL (blue), D2 (red) and D6 (green) array samples depicting separation of samples into time point clusters. PCA was conducted using normalized probe intensities from all differentially expressed genes identified in time point-based microarray analysis (n=2456 probes/sample). **B.** Analysis of principal component 1 (PC1) scores generated for each sample confirming the separation of samples into time point clusters along the PC1 axis. These findings confirmed that the primary source of variation within our gene expression dataset was time-dependant. Given ARI was the only time-dependant feature consistent across all study groups; we regarded the most variation (i.e. PC1-46.5%) to represent the ARI-associated variation within our gene expression dataset. Mean ± SEM, ** p= <0.01, **** p= <0.0001.

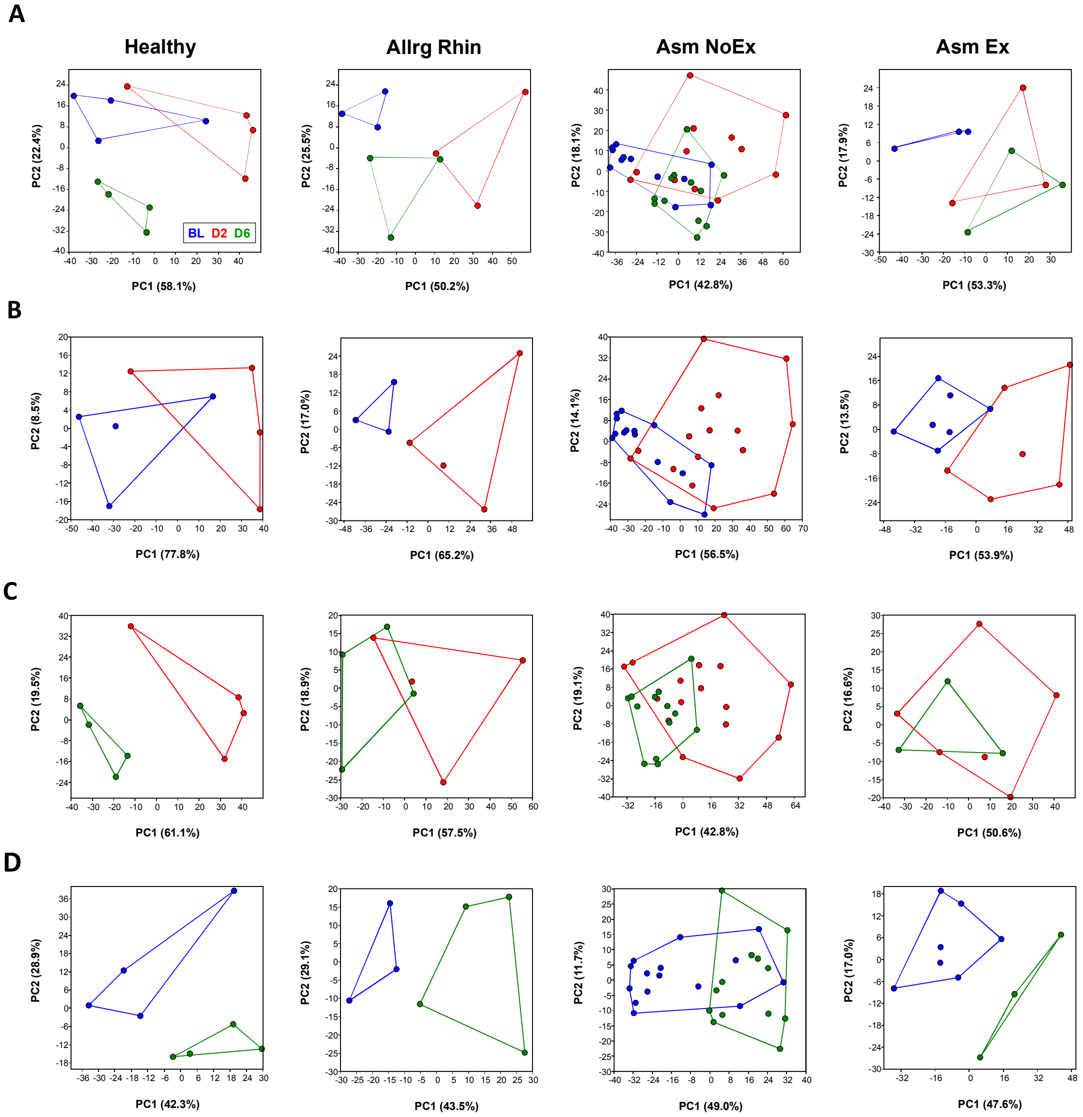

Supplementary Figure S3: Study group-specific principal component analysis (PCA). **A.** Initial PCA using matched BL (blue), D2 (red) and D6 (green) microarray samples from each group indicated overlap between time point clusters predominantly within the asthmatic groups. These results signified the gene expression patterns between specific stages of acute respiratory illness (ARI) were influenced by disease status. **B-D.** Group-specific PCA encompassing three time point transitions of ARI; BL to D2 (**B**), D2 to D6 (**C**) and D6 to BL (**D**). In each case, principal component 1 (PC1) accounted for most variation in data sets and summarized variation driven by ARI. Data generated in each group-specific PCA were subsequently used to determine clarity of transition between time points (PC1 scores, see Supplementary Figure4) and their respective ARI-responsive transcriptional profiles (PC1 loading coefficients). Unmatched microarray samples from each group were included in these additional analyses. Samples are color coded as above. All PCAs were conducted using normalized probe intensities from all differentially expressed genes identified in time point-based microarray analysis (n=2456 probes/sample).

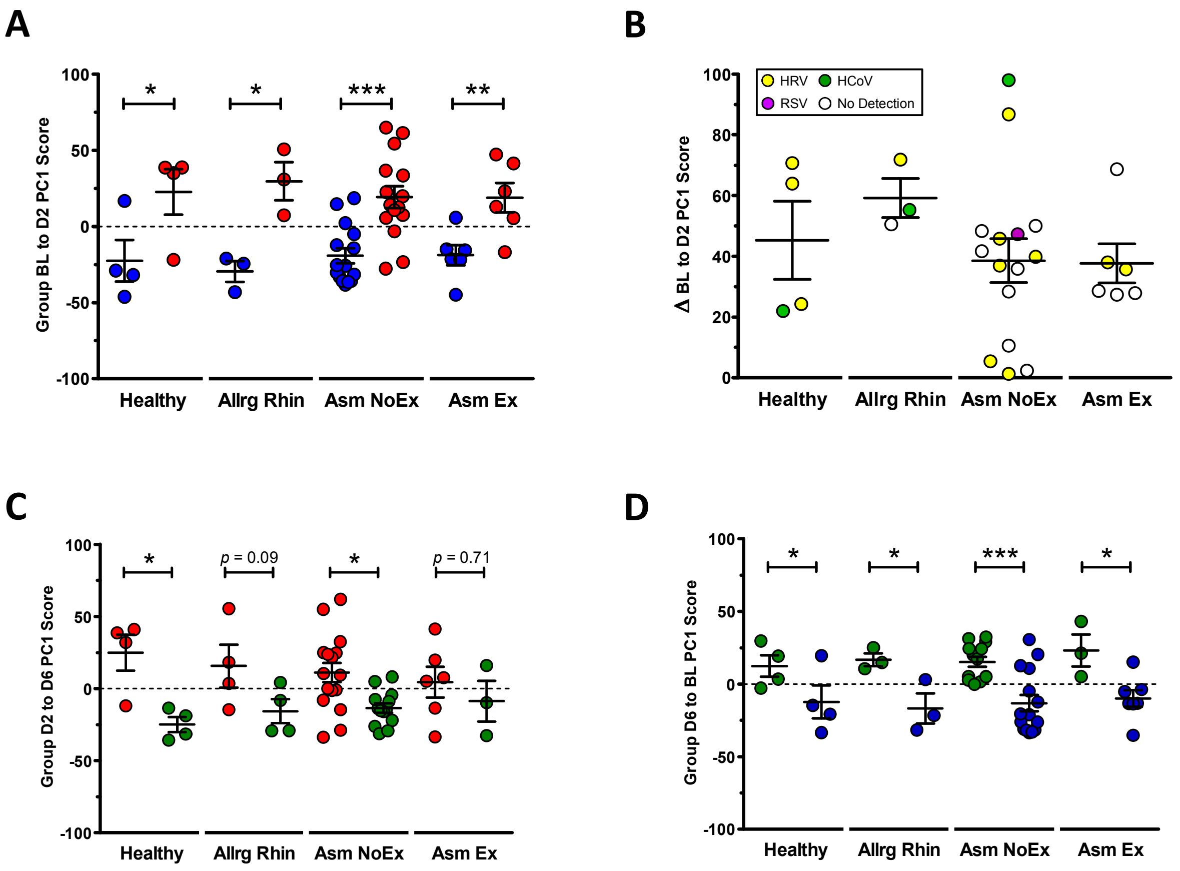

Supplementary Figure S4: Analysis of the principal component 1 (PC1) scores generated in group-specific principal component analysis (PCA, see Supplementary Figure S3). **A**. Analysis of Baseline (BL - blue) to Day 2 (D2-red) transition indicating the separation between samples along the PC1 axis and a clear transition towards the Peak Stage of acute respiratory illness (ARI) in all study groups. **B.** Plot depicting the transition to the Peak Stage was associated predominantly with the presence of respiratory virus within all study groups. The transition towards the Peak Stage was measured by the positive change in PC1 scores between BL and D2 microarray samples. Circles represent individual participants and are filled based on virus identification data collected at D2. HRV- Human Rhinovirus, HCoV – Human Coronavirus, RSV- Respiratory Syncitialvirus. **C.** Analysis of the D2 to Day 6 (D6) transition indicating the Allrg Rhin and Asm Ex groups failed to exhibit a clear separation between D2 (red) and D6 (green) samples and that disease status influenced the transition towards the Reduction Stage of ARI **D.** Analysis of PC1 scores generated in group specific D6 to BL PCA confirming a clear transition towards the Resolution Stage of ARI for all study groups. Mean ± SEM, * p= <0.05 ** p= <0.01, *** p= <0.001.

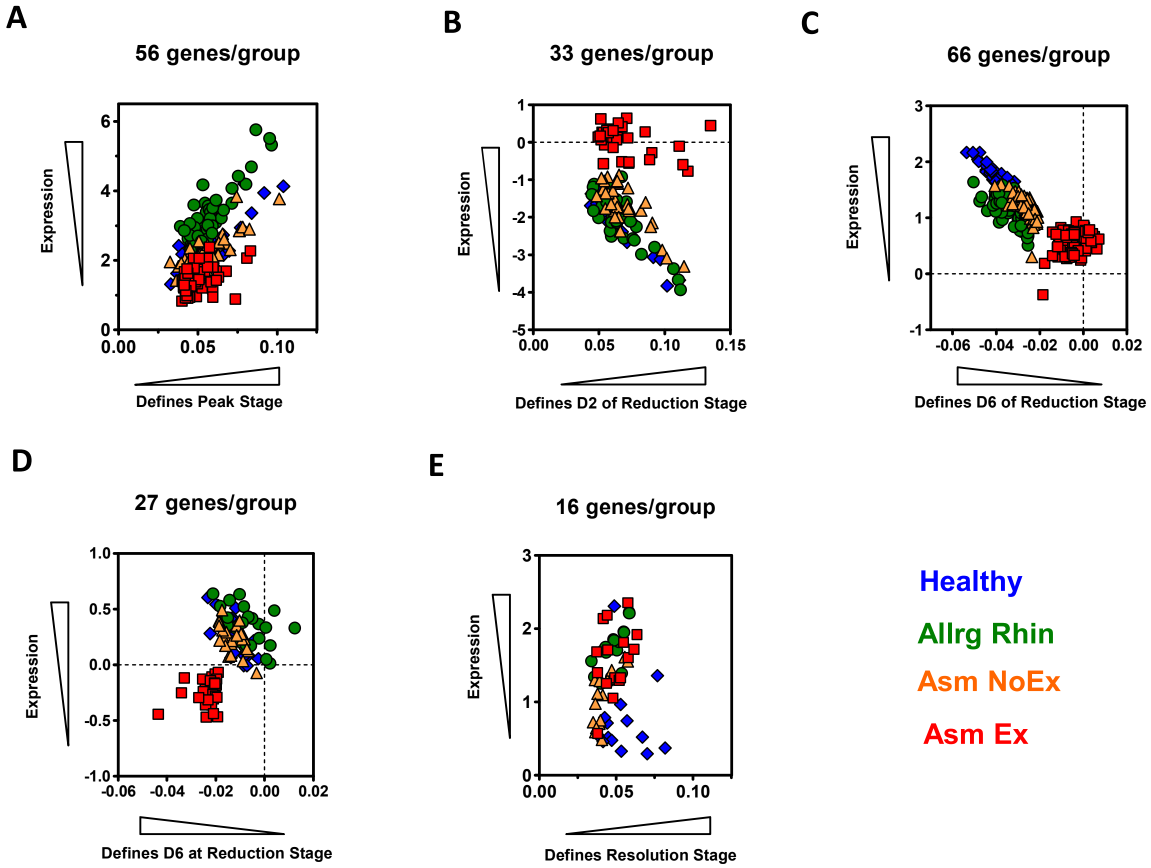

Supplementary Figure S5: Relationship between gene expression and PC1 loadings. Correlation analysis indicated that the presence of underlying disease, particularly asthma exacerbation (red) influences the magnitude and direction of the transcriptional profiles that define each stage of acute respiratory illness (ARI). **A**. Core-D2 genes that defined the Peak Stage. **B**. Core-D2 genes that defined the D2 time point at the Reduction Stage. **C**. Shared-D6 genes that defined D6 of the Reduction Stage. **D**. Unique-D6 genes that defined the D6 time point specifically within the Asm Ex group. **E**. Core-D6 genes that defined the Resolution Stage. Results
